# Genome-wide identification and expression analysis of *auxin response factors* in peanut (*Arachis hypogaea* L.)

**DOI:** 10.7717/peerj.12319

**Published:** 2021-10-21

**Authors:** Peipei Li, Qian Ma, Chengxin Qu, Shuliang Zhu, Kunkun Zhao, Xingli Ma, Zhongfeng Li, Xingguo Zhang, Fangping Gong, Dongmei Yin

**Affiliations:** Henan Agricultural University, College of Agronomy & Center for Crop Genome Engineering, Henan Agricultural University, Henan, China, Zhengzhou, Henan Province, China

**Keywords:** Pod development, Auxin response factors, Auxin, Evolutionary analysis, Hormonal response

## Abstract

Auxin response factors (ARFs) are transcription factors that regulate the expression of auxin response genes, and have important functions in plant growth and development. In this study, available genome data for peanut (*Arachis hypogaea* L.) were used to identify *AhARF* genes. In total, 61 *AhARFs* and 23 *AtARFs* were divided into six groups (I–VI). Molecular structural analysis revealed that the protein members of AhARF contain at least two domains, the B3 domain and the Auxin-resp domain, and that some have a C-terminal dimerisation domain. Screening of the transcriptome data of 22 tissues of *A. hypogaea* cv. Tifrunner in a public database showed high expression levels of *AhARF2* and *AhARF6*. *AhARF6* was expressed more highly in the stem and branch than in the root and leaf of the wild species *Arachis monticola* (*A. mon*) and cultivated species H103. After treatment with exogenous auxin (NAA), the expression of *AhARF6* was inhibited, and this inhibition was greater in *A. mon* than in H103. The transcriptome map revealed that the expression of *AhARF6* was higher in the larger pods of H8107 and ZP06 than in the medium pods of H103 and small pods of *A. mon*. Moreover, *AhARF6*-5 was proven to be localised in the nucleus, consistent with the location of *AtARF6*. These results suggest that *AhARF6* may play an important role in pod development in peanut.

## Introduction

Auxins play a very important role in the regulation of plant growth and development processes (De Smet & Jurgens, 2007; Jain & Khurana, 2009; Zhao, 2010), such as vascular elongation, embryogenesis, lateral root initiation, flower and fruit development, and apical dominance. Auxins can regulate the activity of auxin response factors (ARFs) (Guilfoyle & Hagen, 2007; Okushima et al., 2005; Yang et al., 2013), which are transcription factors that regulate the expression of auxin response genes. To our knowledge, ARFs were first reported in *Arabidopsis thaliana* (Ulmasov et al., 1997), and the ARF gene family was subsequently identified in *Arabidopsis*. A typical ARF consists of a highly conserved N-terminal B3-like DNA-binding domain (B3 domain, PF02362, InterPro entry IPR003340) and a C-terminal dimerisation domain. The middle region (Auxin-resp domain, PF06507, InterPro entry IPR010525), which is located between the B3 domain and C-terminal dimerisation domain, has been proposed to function as an activation or repression domain (Tiwari, Hagen & Guilfoyle, 2003; Ulmasov, Hagen & Guilfoyle, 1999). A previous study indicated that ARFs also have a C-terminal convergent functional domain Aux_IAA (Hagen & Guilfoyle, 2002). *ARFs* can specifically combine with the auxin response element (AuxRE) “TGTCTC” in the promoter region of auxin response genes to regulate them.

*ARFs* have been identified in many species, including *Arabidopsis* (Okushima et al., 2005), tomato (*Solanum lycopersicum*) (Wu et al., 2011), rice (*Oryza sativa*) (Wang et al., 2007), corn (*maize*) (Xing et al., 2011), soybean (*Glycine max*) (Chien et al., 2013), populus (*Populus trichocarpa*) (Kalluri et al., 2007), rape (*Brassica napus)* (Li et al., 2019), and litchi (*Litchi chinensis* Sonn.) (Zhang et al., 2019a). In *Arabidopsis*, *ARFs* play a vital role in plant growth and development processes, especially root development, leaf aging, flower formation, and fruit development (Goetz et al., 2007; De et al., 2008; Wu et al., 2020). In potato (*Solanum tuberosum* L.) crown bud, the expression level of *StARF6* was higher around the apical meristem than in other tissues, suggesting that *StARF6* is related to tissue growth and vascular bundle development (Wu, Tian & Reed, 2006). *AtARF6* and *AtARF8* are homologous genes in *Arabidopsis* that have been proven to have similar functions (Nagpal et al., 2005). Both *AtARF6* and *AtARF8* are involved in regulating plant growth and flower maturation. Double or single mutants of *arf6* and/or *arf8* have similar phenotypes, such as delayed flower maturation, the appearance of small petals, short stamens, and delayed anther release (Nagpal et al., 2005). Previous studies have shown that *ARF6* and *ARF8* regulate the jasmonic acid (JA) biosynthetic pathway by inhibiting *KNOX* gene expression. *ARF6* and *ARF8* promote JA synthesis, which induces the expression of the *MYB21* and *MYB24* required for the growth of stamens and pistils during flowering (Finet et al., 2010; Nagpal et al., 2005; Reeves et al., 2012; Tabata et al., 2010). Genes belonging to the *ARF6* subfamily are targeted by microRNA167. Overexpression of microRNA167 in transgenic tomato plants inhibited the expression of *SlARF6* and *SlARF8*, leading to organ development defects in tomato flowers (Liu et al., 2014; Wu et al., 2011). An optical signal can inhibit the DNA-binding ability of ARF through direct interactions among the photoreceptors *CRY1*, *phyB*, and *ARF* (Mao et al., 2020), and then inhibit auxin signal transduction and induce hypocotyl elongation in *Arabidopsis*. *SlARF7* is expressed highly in unpollinated mature ovaries. Moreover, studies have shown that the transcript level of *SlARF7* increases during tomato flower development, is maintained at a constant high level in mature flowers, and decreases within 48 h after pollination, suggesting that *SlARF7* regulates fruit setting and development (De et al., 2008).

Peanut (*Arachis hypogaea* L.) is a major oil crop grown worldwide. Seeds of peanut have a high oil content and abundant protein. Peanut is an allotetraploid with 40 chromosomes (AABB = AA × BB, 2n = 4x = 40). The genome sequences of the peanut tetraploid cultivated species *A. hypogaea* cv. Tifrunner (Bertioli et al., 2019), *A. hypogaea* var. Shitouqi (Zhuang et al., 2019), and the two diploid wild species *A. duranensis* and *A. ipaensis* (Bertioli et al., 2016; Chen et al., 2016; Lu et al., 2018; Yin et al., 2020) have been completed. The genome sequence of the wild allotetraploid species *A. monticola* (*A. mon*) has also been completed (Yin et al., 2018). *A. mon* plays an important role in the domestication of peanut, from the wild diploid to cultivated tetraploid species (Yin et al., 2020). Extensive data provide a strong foundation for study of the specific gene families of peanut. In this study, the genome version of *A. hypogaea* var. Tifrunner was used to identify ARF gene family members. Comprehensive information about the ARF family in peanut, including chromosome locations, phylogenetic relationships, expression patterns, and gene structures, is provided here. The results may facilitate functional studies of the *AhARF* gene family in peanut.

## Materials & Methods

### Identification of peanut ARF gene family

*A. hypogaea* Tifrunner version 1.0 was used here. First, the amino acid sequences of *AtARFs* were downloaded from the *Arabidopsis* Information Resource (TAIR9, https://www.arabidopsis.org/). Then, homologs of peanut were searched in PeanutBase (https://peanutbase.org/) using BlastP (*P* = 0. 001) according to the amino acid sequences of *AtARFs*. Second, BlastP searches of the ARF domain “PF06507” were performed on the PeanutBase website to find *ARF* genes. The results of the two searches were integrated and redundant genes were discarded. Subsequently, through a domain analysis performed using a hidden Markov model (HMM) (http://pfam.xfam.org/), we selected sequences with *P* < 0.001, and then deleted incomplete and redundant amino acid sequences. We identified 62 ARFs in *A. hypogaea* Tifrunner, 28 in *A. ipaensis*, 27 in *A. duranensis*, and 33 in *A. mon*. Each ARF contained a B3 domain (PF02362) and Auxin-resp domain (PF06507). Among these, there was one gene without a chromosomal location in the family of Tifrunner, which was deleted. Finally, 61 *AhARF* gene family members were identified in Tifrunner, and the *AhARF* genes were named *AhARF1-1* to *AhAR*F19-5 according to their annotation and chromosome location (Additional File 2: Table S1). For example, *AhARF6-5* was annotated as auxin response factor 6 with fifth rank according to position on all of the chromosomes of the AhARF6 subfamily.

### Sequence analyses and the construction of evolutionary tree

The whole proteins sequences of 61 AhARFs and 23 AtARFs (Additional file 2: Table S5) were performed using ClustalW in the software of MEGA-X (https://www.megasoftware.net/). Then, the above results were used to construct an unrooted evolutionary tree by Maximum Likelihood method (1,000 bootstrap replications).

### Chromosomal distribution and tandem duplication analysis of *AhARF* genes

The aforementioned AhARFs were used to screen ARFs in the *A. ipaensis K30076 1.0, A. duranensis V14167 1.0*, and *A. mon* genome databases (Yin et al., 2018). The location of each *AhARF* gene was identified according to the physical location information of the peanut genome annotated by *A. hypogaea* Tifrunner 1.0. According to the amino acid sequence of AhARF from Tifrunner, the similarity for repeated sequence analysis using the BLAST method was set to 90%. Finally, tandem duplication analysis was performed and chromosomal location diagrams were generated using the program Circos-0.69-6 (http://circos.ca) (Krzywinski et al., 2009).

### Expression patterns of *AhARF* genes in different tissues of Tifrunner

The transcription levels of *AhARF* genes in different tissues of Tifrunner were obtained from a public database (https://peanutbase.org/). At 10 days post-emergence, 22 types of tissues were sampled (Clevenger et al., 2016). The samples included seedling leaf, main stem leaf, lateral stem leaf, vegetative shoot tip, reproductive shoot tip, root, nodules, perianth, gynoecium, androecium, aerial gynophore tip, subterranean gynophore tip, Pattee 1 pod, Pattee 1 stalk, Pattee 3 pod, Pattee 5 pericarp, Pattee 5 seed, Pattee 6 pericarp, Pattee 6 seed, Pattee 7 seed, Pattee 8 seed, and Pattee 10 seed (Additional File 2: Table S2). Fragments per kilobase million (FPKM) data were used here. TBtools software (Chen et al., 2020) was applied to visualise these publicly available data. A heatmap was constructed with ClustalW and TreeView using the average data.

### Proteins structure analysis of ARF6 sub-family

Total 16 amino acid sequences of ARF6, including peanut (six ARF6 sub-family amino acid sequences), corn (one ARF6), rice (two ARF6), tomato (two ARF6), *Arabidopsis* (one ARF6), soybean (one ARF6), Medicago (two ARF6), *Lupinus micranthus Guss* (one ARF6) were obtained from NCBI. DNAMAN was used to align the ARF6 amino acid sequences of peanut and other species, and HMMER was used for domain prediction. The AhARF6 sequence motifs were identified using the MEME program (http://meme-suite. org/tools/meme), and *AhARF6* gene structures were deduced using the Gene Structure Display Server (GSDS2, http://gsds.cbi.pku.edu.cn/). TBtools software was used to combine the result of evolutionary tree, domain and conserved motif of ARF6. AhARF6 proteins properties were analyzed, including the number of amino acids, molecular weight, theoretical pI, instability index, aliphatic index, grand average of hydropathicity (GRAVY), and subcellular localization prediction by using ProtParam (https://web.expasy.org/protparam/).

### Transcriptomics analysis of four peanut varieties with different pod/seed sizes

Four peanut varieties with different pod/seed sizes were used here. Among these, ZP06 and H8107 are large-pod varieties, H103 is a medium-pod variety, and *A. mon* is a small-pod variety. These varieties were planted in the same area (Henan, China). The size of the planted area was 0.34 ha. Pod samples for further experiments were collected at different specific developmental stages (15, 35, 55, and 75 days after flowering). Pods were manually separated into shells and seeds. The shells and seeds for each developmental stage of each variety were mixed, immediately frozen in liquid nitrogen, and stored at −80 °C for RNA extraction and subsequent transcriptomics analysis. Three replications were performed. FPKM values of *AhARF6* subfamily members were collected to evaluate the expression levels of the different varieties. A heatmap of *AhARF6* was generated using TBtools.

### Construction of GFP vector and subcellular localization of *AhARF6-5* gene

To investigate the subcellular localization of the AhARF6-5 protein, we transiently expressed green fluorescent protein (GFP) with AhARF6-5 protein in tobacco (*Nicotiana benthamiana*) leaf epidermal cells. The CDS of *AhARF6*-5 was cloned using Phanta Max Super-Fidelity DNA Polymerase (P505; Vazyme, Nanjing, China) and divided into three segments. Three pairs of homologous recombination primers (Additional file 2: Table S3) were designed using Vazyme’s official website (https://crm.vazyme.com/cetool/simple.html). The amplification products were cloned using the ClonExpress Ultra One Step Cloning Kit (C115, Vazyme, Nanjing, China) and ligated into the PFGC5941-35S-GFP (35S-GFP) vector. The recombined plasmids were then transformed into *Agrobacterium tumefaciens* strain EHA105; transient expression and infiltration were performed using previously published protocols (Sparkes et al., 2006; Zhang et al., 2019b). Leaves transformed with the 35S-GFP vector alone were used as the controls. Fluorescence and bright-light images of transiently infected tobacco leaves were obtained 48–72 h after infiltration by using a laser scanning confocal microscope (LSM710; Axio Observer Z1, Zeiss, Jena, Germany).

### Plant materials and hormone treatments

H8107, ZP06 and H103 are tetraploid cultivated species, and *A.mon* is tetraploid wild species. Mutation identification of each member of *AhARF6* sub-family was done in four varieties. The cultivar H8107, ZP06 and H103 were developed in our laboratory. Here, H103 and *A.mon* were selected to be materials corresponding to cultivated species and wild species of peanut. For hormone treatments, six seedlings of peanut were grown in 1/2 Hoagland solution in a light incubator under a 16 h photoperiod (32 °C) and 8 h dark (25 °C) period. When *A.mon* and H103 were grown up to fifth leaf stage, 100 µM NAA was subjected to their leaves. Around 100 mg of the roots, leaves, stems, and branches were sampled at 2, 4, 8, and 12 h after NAA treatment. Control was collected without NAA treatment at the same stage, immediately frozen in liquid nitrogen, and then stored at −80 °C until RNA extraction. Three replications were done.

### Quantitative reverse transcription polymerase chain reaction (qRT-PCR)

RNA was extracted from the roots, stems, leaves, and branches by using TransZol Plant (TaKaRa, Dalian, China). The RNA concentration and quality were evaluated with a NanoDrop One spectrophotometer (Thermo Fisher Scientific, Madison, WI, USA) and standard agarose gel electrophoresis (1%, w/v). The RNA was used for the reverse transcription of cDNA with EasyScript One-Step gDNA Removal and CDNA Synthesis SuperMix (TaKaRa, Dalian, China). The primers were designed using the Real-time PCR (TaqMan) Primer and Probes Design Tool website (https://www.genscript.com/tools/real-time-pcr-taqman-primer-design-tool). The total PCR volume was 20 µL, and it contained 0.4 µL of the forward primer, 0.4 µL of the reverse primer, two µL of cDNA, 10 µL of TransStart Top Green qPCR SuperMix (TaKaRa), and 7.2 µL of nuclease-free H_2_O. TB Green Premix ExTaq II (Tli RNaseH Plus) Mix (TaKaRa, Dalian, China) was used for qRT-PCR on the CFX96 Touch real-time PCR system (Bio-Rad, Hercules, CA, USA). The PCR protocol was as follows: 95 °C for 30 s, followed by 40 cycles of 95 °C for 5 s and 60 °C for 30 s. Each reaction biology experiments were repeated three times, and the 2^−ΔΔCT^ (Livak & Schmittgen, 2001) method was used to calculate the relative gene expression levels of *AhARF6* genes in peanut. The primers for qRT-PCR are listed in Additional file 2: Table S4. *AhActin* (EG030635) was amplified as endogenous controls for qRT-PCR *(Reddy et al., 2013*). The specific primers of *AhARF6* sub-family members used to detect transcripts are listed in (Additional file 2: Table S4).

## Results

### Identification of peanut *ARF* gene family

A total of 61 *AhARF* genes were identified and named *AhARF1-1* to *AhARF19-5* (Additional File 2: Table S1) according to their annotation and chromosome location in PeanutBase (https://peanutbase.org/). All AhARFs contained B3 and Auxin-resp domains, and some of them had the Aux_IAA domain.

### Phylogenetic analysis of *ARF* genes

**Figure 1:**
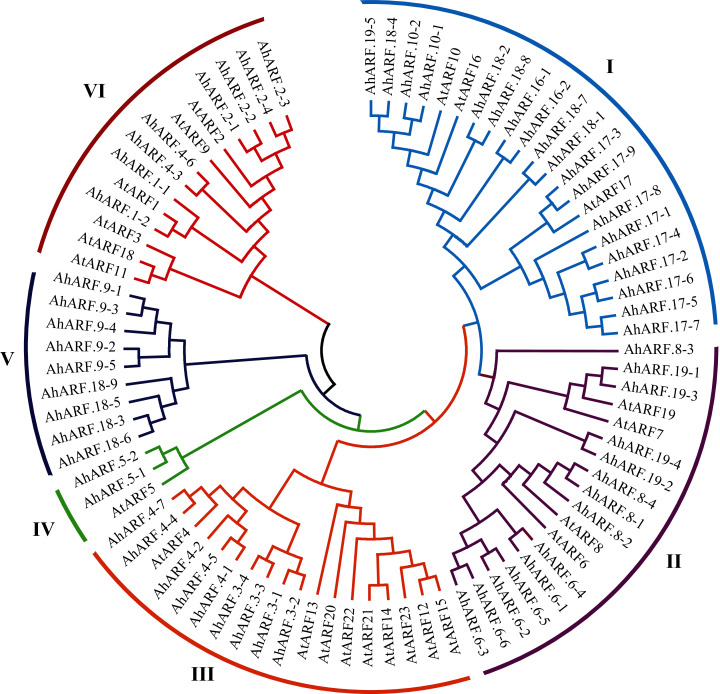
Phylogenetic analysis of auxin response factor (ARF) proteins from peanut and *Arabidopsis*. The phylogenetic tree was constructed using the maximum likelihood method in MEGA-X. The numbers represent the confidence of the branches.

Phylogenetic analysis of the ARF proteins from peanut and *Arabidopsis* was conducted to study the evolutionary relationships (Additional File 2: Table S5). The results demonstrated that AhARF proteins can be divided into six subgroups according to the clades and classifications for *Arabidopsis* (Fig. 1). Group I contains the highest number of ARF proteins (*n* = 22: 19 AhARF proteins from peanut and three from *Arabidopsis*). Group II has 18 ARF proteins (14 AhARF proteins from peanut and 4 AtARF proteins from *Arabidopsis*). Group VI has 14 ARF proteins (eight AhARF proteins from peanut and six from *Arabidopsis*). These results indicate that most peanut AhARF proteins have high homology with *Arabidopsis* AtARF proteins. Class A ARFs (Cédric et al., 2013), including ARF5, ARF6, ARF7, ARF8, and ARF19, are transcriptional activators. Among these, AhARF6/AtARF6, AhARF8/AtARF8, and AhARF19/AtARF19 belong to Group II, and AhARF5/AtARF5 belong to Group IV. There is no AhARF7 in peanut. The results show that many peanut *ARF* genes have high similarity with *ARF* genes of *Arabidopsis*.

### Chromosomal distribution and tandem duplication analysis of *AhARF* genes

**Figure 2:**
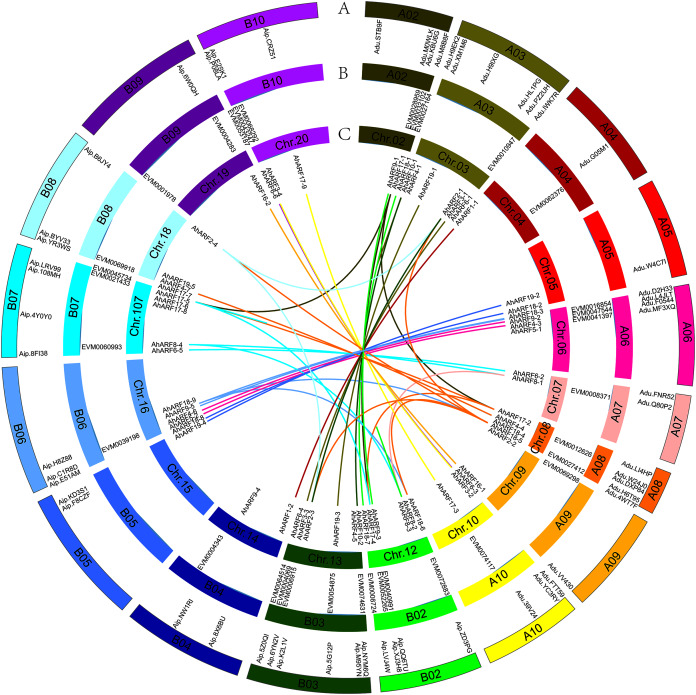
Distribution and synteny analysis of *AhARF* gene family on peanut chromosomes. Distribution and synteny analysis of *AhARF* gene family on peanut chromosomes. The approximate chromosomal locations of the *AhARF* genes are indicated on the periphery. The colored lines linking genes from different chromosomes denote segmental duplication events. A. *A. duranensis* (diploid wild species) and *A. ipaensis* (diploid wild species). B. *A. mon* (tetraploid wild species). C. *A. hypogaea* (tetraploid cultivated species). Two genes on the same curve are a couple of segmentally duplicated genes at *A. hypogaea*.

Based on their annotated genomic locations, the 61 *AhARF* genes were found to be widely distributed among the peanut chromosomes (except for chromosomes 01 and 11) (Fig. 2). The number of genes on homologous chromosomes was not exactly the same. Chromosome 3 contained the most *AhARF* genes (*n* = 7). Chromosomes 12, 13, and 17 had six genes. Chromosome 8 had five *AhARF* genes. Chromosomes 6 and 16 had four genes. Chromosomes 10, 18, and 20 possessed three genes. Chromosomes 5, 14, and 15 had two genes, and chromosomes 4, 9, and 19 had only one.

Duplication (fragment and tandem duplication) is important in the analysis of the evolution of gene families. Twenty-six segmental duplication events have occurred according to the tetraploid *A. hypogaea* peanut amino acid sequence (Fig. 2): *AhARF6-2* to *AhARF6-5*, *AhARF6-3* to *AhARF6-6*, *AhARF6-1* to *AhARF6-4*, and so on (Fig. 2). No tandem duplication was observed. The results suggest that only segmental duplication took part in the evolution of the peanut *AhARF* gene family. The number of *ARF* genes was identified; there were 28 in *A. ipaensis*, 27 in *A. duranensis*, 33 in *A. mon*, and 61 in *A. hypogaea*. There is no evolutionary rule regarding the different numbers of *ARF* genes in diploid wild species, tetraploid wild peanut, or tetraploid cultivated peanut, possibly because the genome of *A. mon* has not been completely assembled.

### Expression pattern analysis of *AhARF* genes in different tissues

To understand the functions of *AhARF* genes in peanut, we investigated the transcription levels of *AhARF* genes in different tissues by using publicly available transcriptome datasets (Clevenger et al., 2016). Hierarchical clustering analysis was performed, and heatmaps were generated to display the expression patterns of the *AhARF* genes (Additional File 1: Fig. S1) by using TBtools. *AhARF6* genes were expressed in all tissues of peanut. The expression levels of *AhARF6* were highest in stamen, stem, and branch, followed by fruit needle and pod; the expression levels were relatively low in the other parts. However, *AhARF6-2* and *AhARF6-5* were expressed at lower levels than the other four *AhARF6* subfamily genes in all tissues. These results suggest that *AhARF6* genes may play a role in flower organ development, tissue organ growth, and pod maturation. The *AhARF2* subfamily was expressed highly in all tissues, suggesting key roles in tissue development. The expression levels of other *ARF* genes, especially *AhARF3*, *AhARF4*, *AhARF10*, *AhARF17*, and *AhARF18*, were very low or even absent. Different *AhARF* genes may play different roles in growth and tissue development in peanut. Understanding the expression patterns of *AhARF* genes in different tissues can provide a foundation for identifying the functional genes of peanut.

### AhARF6 protein properties and sequence analyses of ARF6 in peanut and other plants

Analysis of the protein properties of AhARF6 (Additional File 2: Table S7) showed that the length and molecular weight of AhARF6 proteins are relatively similar, ranging from 810–923 amino acids and 89.83–101.67 kDa, respectively. The theoretical isoelectric point varied from 4.13 to 11.38. All AhARF6 proteins were considered unstable, because the instability index was higher than 40 (between 61.49 and 69.08). The aliphatic index of the AhARF6 proteins was predicted to range from 72.70 to 76.57. Because of a relatively low average hydrophilic value (<0), all AhARF6 proteins were predicted to be hydrophilic. Subcellular localisation analysis showed that all AhARF6 proteins are localised in the nucleus, indicating that AhARF6 may play a role.

**Figure 3:**
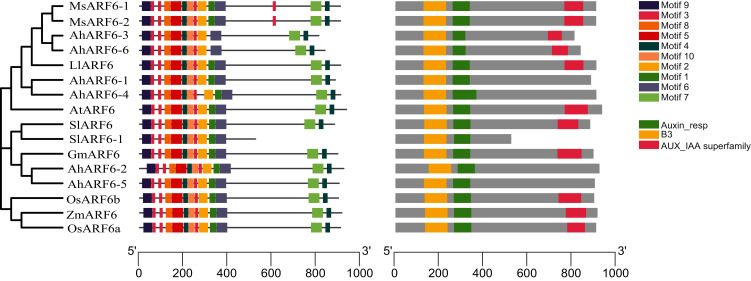
ARF6 protein sequence analyses of peanut, corn, rice, *Arabidopsis*, soybean, tomato, medicago and *Lupinus micranthus Guss*. (A) Phylogenetic tree was constructed using MEGA-X with the NJ method. Corn, *ZmARF6*. Rice, *OsARF6*. Peanut, *AhARF6*. *Arabidopsis thaliana*, AtARF6. Soybean, *GmARF6*. Tomato, *SlARF6*. Medicago, MsARF6. *Lupinus micranthus Guss*, LmARF6. (B) Motif distribution of ARF6 proteins. Different motifs are indicated by different colors. The sequence information for each motif is provided in Additional file 2: Table S8. (C) Structural analysis of ARF6 proteins.

To investigate the structural features of *ARF6* proteins, 16 ARF6 amino acid sequences of *Arabidopsis*, maize, tomato, rice, soybean, medicago, and *Lupinus micranthus* Guss. were obtained from the National Center for Biotechnology Information (NCBI; https://www.ncbi.nlm.nih.gov/). The ARF6 evolution analysis (Fig. 3A) showed that AhARF6 had the highest similarity with MsARF6, LmARF6, and GmARF6, indicating that peanut is most closely related to medicago, *Lupinus micranthus* Guss., and soybean. AhARF6 had the lowest homology with maize and rice. Ten conserved motifs (Fig. 3B) (motifs 1–10) were identified. Most of the motifs are located within the N-terminal region; very few (motifs 4 and 7) are within the C-terminal region. Eleven of the ARF6 protein sequences (Fig. 3C) contain the B3, Auxin-resp, and Aux_IAA domains, and five protein do not have the Aux_IAA domain. In this phylogenetic tree, closely related *ARF6* genes showed a conserved intron–exon structure and similar motifs in terms of alignment, which suggests that ARF6 members with similar structures clustered along the same branch may have similar biological functions.

### Analysis of pod expression of the *AhARF6* subfamily in four varieties and subcellular localisation of *AhARF6-5* gene

**Figure 4:**
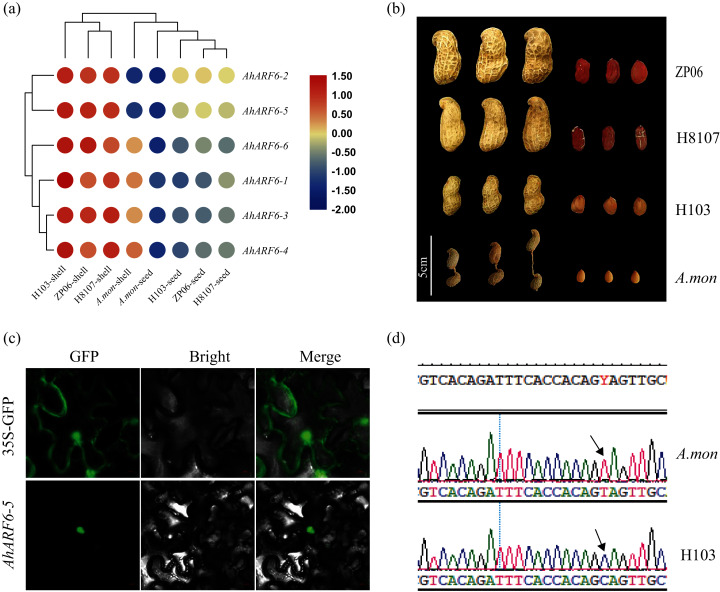
Heatmap for pod expression in four varieties and subcellular localization of AhARF6-5 proteins. Heatmap for pod expression in four varieties and subcellular localization of AhARF6-5 proteins. (A) Peanut pod and seed phenotype of ZP06, H8107, H103 and *A.mon*. (B) Expression analysis of seeds of ZP06, H8107, H103 and *A. mon* by transcriptome analysis. Bar showed log_2_ (FPKM). (C) Mutation identification of *AhARF6-5* gene in *A. mon* and H103. (D) Subcellular localization of AhARF6-5 proteins in tobacco leaves.

To understand the function of the *AhARF6* gene subfamily in peanut, we measured its expression levels in four pod varieties (Additional File 2: Table S9). The FPKM data were retrieved from the transcriptome data of the four varieties: H8107, ZP06, H103, and *A. mon*. A heatmap was constructed from the results (Fig. 4A). All *AhARF6* subfamily genes were expressed in the pods, but the expression levels varied. The expression of *AhARF6* was significantly higher in pod shell than seed. All six *AhARF6* genes were more highly expressed in the shell of H103 than in the shell of the other three varieties. Additionally, the expression in shell and seed was lower in *A. mon* than in the other samples. In seed, *AhARF6* showed higher expression in large-pod cultivars (ZP06 and H8107) than in the medium-pod cultivar (H103), and it showed the lowest expression in the super-small pod *A. mon* (Figs. 4A, 4B). In summary, *AhARF6* expression appears to be associated with the trait of pod size. Quantitative trait locus (QTL) or other types of analysis are needed to better understand the regulation of fruit size by AhARF6.

Subcellular localisation analysis of AhARF6-5 was performed. Confocal laser microscopy revealed green fluorescent protein (GFP) in the nucleus (Fig. 4C), demonstrating that the AhARF6-5 protein is localised in the nucleus. Mutation identification of each member of the *AhARF6* subfamily was done for all four varieties. In cultivated species H103 and tetraploid wild species *A. mon*, *AhARF6-5* has a C > T mutation in the coding region (Fig. 4D), which results in early termination of translation in *A. mon*. Auxin transcription factors were expressed in the nucleus and regulate downstream genes (Tiwari, Hagen & Guilfoyle, 2003).

### Expression of *AhARF6* genes in different tissues

**Figure 5:**
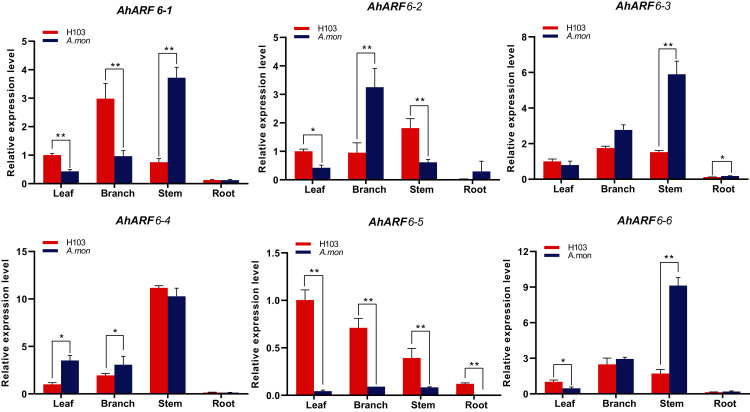
Expression analysis of *AhARF6* sub-family genes in different tissues. qRT-PCR analysis of *AhARF6* transcript levels in leaves, roots, stems, and branches was performed. Values are means ± SD (*n* = 3). Significant difference between H103 and *A. mon* at *P* < 0.05 and *P* < 0.01 (two sample t-test) is denoted by * and **, respectively.

We investigated the expression levels of *AhARF6* genes in four tissues of *A. mon* and H103: leaves, branches, stems, and roots at the fifth leaf stage. The different materials were applied for quantitative reverse transcription polymerase chain reaction (qRT-PCR). The results showed that *AhARF6* subfamily genes were expressed in the leaves, branches, stems, and roots of H103 and *A. mon* (Fig. 5), indicating that they are constitutively expressed genes. Further analysis revealed that most of the *AhARF6* genes were expressed most highly in stems, followed by branches, and that the expression levels were lowest in roots (Fig. 5). However, the expression of *AhARF6-5* was higher in leaves than in the other tissues. The expression levels of *AhARF6* genes differed between *A. mon* and H103. In stem, *AhARF6-1*, *AhARF6-2*, *AhARF6-3*, *AhARF6-4*, and *AhARF6-6* were expressed at levels 1.06–4.95 times higher in *A. mon* than in H103. These results indicate that *ARF6* genes are mainly expressed in branches and stems, and that the expression pattern of *AhARF6-5* is different from the other five *AhARF6* genes. In summary, *AhARF6* may play key roles in the processes of peanut pod/seed growth and development.

### Early responses of *AhARF6* to exogenous NAA treatments

**Figure 6:**
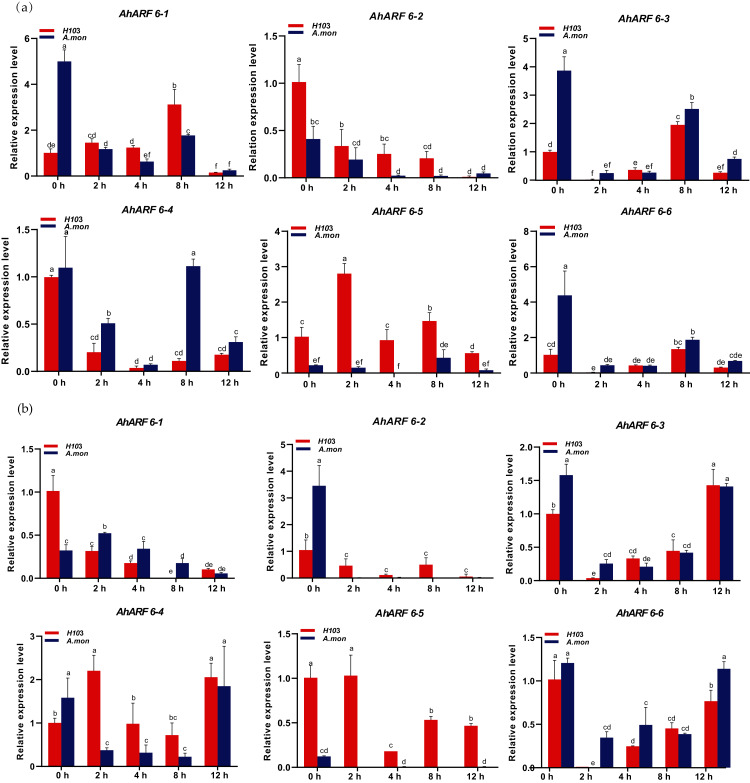
Expression analysis of *AhARF6* sub-family genes in response to NAA treatment in peanut stem and branch. qRT-PCR analysis of *AhARF6* transcript levels was performed at the fifth leaf stage of plants treated with NAA for 0, 2, 4, 8 h. (A) Expression in the stem. (B) Expression in the branch. Values are means ± SD (*n* = 3). Different lowercase letters denote significant differences between the any two stages (*P* < 0.05, one-way ANOVA and Tukey’s test for multiple comparisons).

To investigate the early responses of *AhARF6* to exogenous auxin (NAA) application, 100 µM NAA was sprayed, and fifth-stage leaves of H103 and *A. mon* were analysed. The expression levels of *AhARF6-1*, *AhARF6-2*, *AhARF6-3*, *AhARF6-4*, and *AhARF6-6* in the main stem in H103 and *A. mon* initially decreased and then increased gradually over 12 h with NAA treatment (Fig. 6A). However, the expression level of *AhARF6-5* in stem initially increased and then decreased in H103, in contrast to the case of *A. mon*, which is different from the early responses of the other five *AhARF6* genes to NAA. The six *AhARF6* genes were downregulated to various degrees in branch at 4 h after exogenous NAA treatment, but the four *AhARF6* genes (*AhARF6-3, AhARF6-4, AhARF6-5, AhARF6-6*) showed increased expression. *AhARF6-5* exhibited much higher branch expression levels in H103 than in *A. mon* within 12 h of exogenous NAA treatment (Fig. 6B). Exogenous NAA inhibits the expression of *AhARF6* genes, and the expression of *AhARF6* was decreased in the stems and branches of H103 and *A. mon* after treatment (Additional File 2: Table S10). *AhARF6* genes also showed downregulation in leaves and roots (Additional File 1: Figs. S2, S3). The results show that the responses to NAA treatment varied among different tissues.

## Discussion

ARFs are transcription factors specific to plants, and play important regulatory roles in the growth and development of plants. Zhuang et al. (2019) identified 114, 28, and 28 ARF proteins by BLAST in Shitouqi, *A. duranensis*, and *A. ipaensis* using the protein sequence from the *A. hypogaea ARF* gene, respectively; these were grouped into nine clusters according to their maximum likelihood tree. A recent Chinese report identified 62 *AhARF* gene family members, and named them *AhARF1–AhARF62* according to their chromosome location in Tifrunner (Tang et al., 2020). In this study, 61 *ARF* genes (a gene not located on the chromosome was deleted) were identified in Tifrunner and divided into 13 functional subfamilies. Moreover, 27, 28, and 33 members of *AhARF* were found in *A. duranensis*, *A. ipaensis*, and *A. mon*, respectively. This study observed 26 segmental duplication events in 61 ARF proteins based on the criterion of 90% similarity, which is less than the 33 segmental duplication events in 62 ARF proteins identified in a previous report (Tang et al., 2020). The number of *ARF* genes is higher in peanut than in most other plants, such as *Arabidopsis* (23 members) (Okushima et al., 2005), tomato (21 members) (Wu et al., 2011), rice (25 members) (Wang et al., 2007), maize (31 members) (Xing et al., 2011), soybean (51 members) *(Chien et al., 2013*), populus (39 members) (Kalluri et al., 2007), barley (17 members), peach (17 members) (Diao et al., 2020), strawberry (12 members) (Wang et al., 2019), and millet (24 members) (Zhao et al., 2016), and is similar to the number in allopolyploid *B. napus* (67 members) (Wen et al., 2019). The reason for this may be that peanut is an allotetraploid plant, with 40 chromosomes and two sets of independent chromosomes. Thus, the genome of peanut (2.38 Gb) is many times larger than those of *Arabidopsis* and rice (0.125 and 0.466 Gb, respectively). An evolution analysis of the 61 *AhARFs* of peanut and 23 *AtARFs* of *Arabidopsis* showed that they can be divided into six groups, and that eight *AtARFs* are homologous to *AhARFs*. The amino acid sequence analysis of *OsARFs* in rice (Wang et al., 2007) and *AtARFs* in *Arabidopsis* showed that they can be divided into five classes, similar to *ZmARFs* and *AtARFs* (Xing et al., 2011). The results showed that ARF clustering results differ among species.

A recent report on *AhARF* focused on the expression patterns of *AhARF10* (*AhARF3-1* here), *AhARF20* (*AhARF8-1* here), *AhARF23* (*AhARF4-4* here), and *AhARF46* (*AhARF5-2* here) during seed germination and found that played a positive role in the process (Tang et al., 2020). Previous studies on other crops showed that *ARF6* is mainly expressed in the flowers of plants, and is involved in the maturation of flower organs and development of seeds and/or fruits (Kumar, Tyagi & Sharma, 2011; Nagpal et al., 2005). For example, *SlARF8* plays a negative role in controlling fruit size, and the inhibition of *SlARF8* expression enhances tomato fruit development (Goetz et al., 2007). Transgenic plants with decreased *SlARF7* transcription levels formed seedless (parthenocarpic) fruits (De et al., 2008). Previous studies have shown that *ARF8* regulates fruit formation in *Arabidopsis thaliana* and tomato (Goetz et al., 2007). Expression analysis of *CmARF9*, *CmARF16-like*, *CmARF19-like*, *CmARF19*, *CmARF1*, *CmARF2*, *CmARF3*, and C*mARF5* showed that they may be associated with fruit growth in melon during early development (Wu et al., 2020). In Tifrunner, all *AhARF6* genes were expressed most highly in stamens, stems, and branches, followed by fruit needles and pods (Clevenger et al., 2016). Among these, four *AhARF6* genes (*AhARF6-1*, *AhARF6-3*, *AhARF6-4*, and *AhARF6-6*) were expressed at higher levels than *AhARF6-2* and *AhARF6-5* in 22 tissues of peanut. Here, the transcriptome data of H8107, ZP06, H103, and *A. mon* showed that all six *AhARF6* subfamily genes were expressed in pods. Importantly, the large-seed varieties (ZP06 and H8107) showed higher expression of *AhARF6* than the medium-seed variety (H103), and the small-seed *A. mon* showed the lowest expression. These results suggest that *AhARF6* may be related to pod/seed growth and development, although this requires further verification by QTL and other methods.

In *Arabidopsis*, *AtARF6* is mainly expressed in flowers; together with *AtARF8*, it regulates the maturation of flower organs and development of the seed coat (Nagpal et al., 2005; Wu et al., 2011). In tomato, *SlARF6* is expressed in all tissues. *SlARF6A* is expressed more highly in flowers and fruits, and is involved in flower organ development and fruit ripening (Kumar, Tyagi & Sharma, 2011; Maas et al., 2014). In peach, *PpARF6* is expressed in roots, stems, leaves, and flowers, and is involved in fruit ripening (Diao et al., 2020; Li, Ran & Sun, 2016). *FvARF6* is highly expressed in old leaves and might participate in the late growth of strawberry (Wang et al., 2019). We analysed the expression levels of peanut *AhARF6* genes in roots, stems, leaves, and branches and found that they were all expressed in these four tissues, indicating that *AhARF6* genes are constitutively expressed. The expression levels of *AhARF6-1*, *AhARF6-2*, *AhARF6-3*, *AhARF6-4*, and *AhARF6-6* were higher in *A. mon* than H103. *ARF6* is expressed in different tissues in different plants, indicating that *ARF6* may have different functions during the growth and development of different tissues. As well as regulating auxin gene expression, *ARFs* also participate in the regulatory pathways of various hormones, such as gibberellins (O’Neill et al., 2010; Weston, Reid & Ross, 2009), ethylene, abscisic acid, brassinolide (Vert et al., 2008), and salicylic acid. The early expression of *AhARF6* genes following exogenous NAA treatments confirmed that *AhARF6* genes are highly sensitive to auxins. The expression of *AhARF6* genes either decreased or increased after 2 h of treatment with NAA. Most *AhARF6* genes showed a negative response to NAA treatment of the stems and branches. The detailed mechanism of the response should be explored in the near future. An analysis of the AhARF family proteins revealed that all AhARF proteins occur in the nucleus. We also found that 16 ARF6 proteins from different crops have the B3 conserved domain and auxin-resp domain, while some contain the Aux_IAA domain (Tiwari, Hagen & Guilfoyle, 2003; Ulmasov, Hagen & Guilfoyle, 1999). The structure of AhARF6 subgroup proteins is more similar to those of MsARF6, LmARF6, and GmARF6. In this study, *AhARF6-5* had a C > T mutation in the coding region of *A. mon* and H103 (Fig. 4D), which results in the early termination of translation in *A. mon*. According to its expression in the pod of different species, *AhARF6-5* may be involved in the control of pod size.

Basically, *ARFs*, as transcription factors localised in the nucleus, regulate downstream genes. The division of ARF proteins in cells and spatiotemporal expression of *ARF* genes in plants provides an important basis for research on the function of *ARFs* in the processes of plant growth and development. In peach, PpARF4, PpARF6, PpARF10A, and PpARF12 fusion proteins were observed in the nucleus (Diao et al., 2020). The BnARF protein of *Brassica napus* and AtARF protein of *Arabidopsis* are located in the nucleus (Truskina et al., 2021; Wen et al., 2019). Here, subcellular localisation analysis by the transient agroinfiltration method with a GFP fusion construct showed that *AhARF6-5* is located in the nuclear area. The aforementioned results indicate that ARFs are typical nuclear-localised transcription factors. As an important part of the auxin signaling pathway, *ARFs* combine with AuxRE in the auxin response gene promoter to activate or inhibit auxin response gene expression and regulate auxin synthesis (Tiwari, Hagen & Guilfoyle, 2003). *AhARF6-5* is a Class A *ARF*, and *AhARF6-5* is a transcription activator of auxin gene expression. Auxin regulates cell division and elongation, and also determines the size of plant fruit (Perrot-Rechenmann, 2010; Woodward & Bartel, 2005). All of the data from cell culture studies indicate that auxin is a permissive signal of cell division that enables initiation of the cell cycle. *AhARF6* may control peanut pod size by accommodating the expression of downstream auxin genes.

## Conclusions

In this study, 61 *AhARF* genes were identified in the peanut genome and subsequently divided into six groups with 23 *AtARF*s. The AhARF6 subfamily consists of constitutively expressed genes expressed in leaves, stems, branches, and roots. The expression of *AhARF6* in stems and branches of H103 and *A. mon* was decreased after NAA treatment. Exogenous NAA inhibits the expression of *AhARF6* genes. The expression levels of *AhARF6* in shell and seed were lower in *A. mon* than H103, and our findings showed that *AhARF6* genes may play a role in pod/seed development.

## Supplemental Information

10.7717/peerj.12319/supp-1 Supplemental Information 1 Expression patterns of *AhARF* genes in different tissues. The dates are from an expression atlas (Clevenger et al., 2016), Bar showed log_2_ (FPKM) colored blue to red. Information on 22 tissues has been provided in Additional file 2: Table S2. Fragments per Kilobase per Million mapped reads (FPKM) values of the AhARF genes are listed in Additional file 2: Table S6.

10.7717/peerj.12319/supp-2 Supplemental Information 2 Expression analysis of AhARF6 sub-family genes in response to NAA treatment in peanut leaf. qRT-PCR analysis of AhARF6 transcript levels was performed at the fifth leaf stage of plants treated with NAA for 0, 2, 4, 8, and 12 h. Values are means ± SD (*n* = 3). Different lowercase letters denote significant differences between the any two stages (*P* < 0.05, one-way ANOVA and Tukey’s test for multiple comparisons).

10.7717/peerj.12319/supp-3 Supplemental Information 3 Expression analysis of AhARF6 sub-family genes in response to NAA treatment in peanut root. qRT-PCR analysis of AhARF6 transcript levels was performed at the fifth leaf stage of plants treated with NAA for 0, 2, 4, 8, and 12 h. Values are means ± SD (*n* = 3). Different lowercase letters denote significant differences between the any two stages (*P* < 0.05, one-way ANOVA and Tukey’s test for multiple comparisons).

10.7717/peerj.12319/supp-4 Supplemental Information 4 Information of *AhARF* genes.

## Additional Information and Declarations

### Data Availability

The following information was supplied regarding data availability: The raw data are available in the Supplementary Tables.

## References

[ref-1] Bertioli DJ, Cannon SB, Froenicke L, Huang G, Farmer AD, Cannon EKS, Liu X, Gao D, Clevenger J, Dash S, Ren L, Moretzsohn MC, Shirasawa K, Huang W, Vidigal B, Abernathy B, Chu Y, Niederhuth CE, Umale P, Araujo ACG, Kozik A, Do Kim K, Burow MD, Varshney RK, Wang X, Zhang X, Barkley N, Guimaraes PM, Isobe S, Guo B, Liao B, Stalker HT, Schmitz RJ, Scheffler BE, Leal-Bertioli SCM, Xun X, Jackson SA, Michelmore R, Ozias-Akins P (2016). The genome sequences of *Arachis duranensis* and *Arachis ipaensis*, the diploid ancestors of cultivated peanut. Nature Genetics.

[ref-2] Bertioli DJ, Jenkins J, Clevenger J, Dudchenko O, Gao D, Seijo G, Leal-Bertioli SCM, Ren L, Farmer AD, Pandey MK, Samoluk SS, Abernathy B, Agarwal G, Ballen-Taborda C, Cameron C, Campbell J, Chavarro C, Chitikineni A, Chu Y, Dash S, El Baidouri M, Guo B, Huang W, Do Kim K, Korani W, Lanciano S, Lui CG, Mirouze M, Moretzsohn MC, Pham M, Shin JH, Shirasawa K, Sinharoy S, Sreedasyam A, Weeks NT, Zhang X, Zheng Z, Sun Z, Froenicke L, Aiden EL, Michelmore R, Varshney RK, Holbrook CC, Cannon EKS, Scheffler BE, Grimwood J, Ozias-Akins P, Cannon SB, Jackson SA, Schmutz J (2019). The genome sequence of segmental allotetraploid peanut *Arachis hypogaea* L. Nature Genetics.

[ref-3] Chen C, Chen H, Zhang Y, Thomas HR, Frank MH, He Y, Xia R (2020). TBtools: an integrative toolkit developed for interactive analyses of big biological data. Molecular Plant.

[ref-4] Chen X, Li H, Pandey MK, Yang Q, Yu S (2016). Draft genome of the peanut a-genome progenitor (*Arachis duranensis*) provides insights into geocarpy, oil biosynthesis, and allergens. Proceedings of the National Academy of Sciences of the United States of America.

[ref-5] Chien VH, Le DT, Rie N, Yasuko W, Saad S, Thi TU, Keiichi M, Nguyen VD, Kazuko YS, Kazuo S (2013). The auxin response factor transcription factor family in soybean: genome-wide identification and expression analyses during development and water stress. DNA Research.

[ref-6] Clevenger J, Chu Y, Scheffler B, Ozias-Akins P (2016). A developmental transcriptome map for allotetraploid *Arachis hypogaea*. Frontiers in Plant Science.

[ref-7] Cédric F, Annick BD, Scutt CP, Ferdinand M (2013). Evolution of the *ARF* gene family in land plants: old domains, new tricks. Molecular Biology & Evolution.

[ref-8] De Smet I, Jurgens G (2007). Patterning the axis in plants-auxin in control. Current Opinion in Genetics & Development.

[ref-9] De JM, Wolters-Arts MR, Mariani C, Vriezen WH (2008). The solanum lycopersicum auxin response factor 7 (*SlARF7*) regulates auxin signaling during tomato fruit set and development. Plant.

[ref-10] Diao DH, Hu X, Guan D, Wang W, Yang HQ, Liu YP (2020). Genome-wide identification of the *ARF* (auxin response factor) gene family in peach and their expression analysis. Molecular Biology Reports.

[ref-11] Finet C, Fourquin C, Vinauger M, Berne-Dedieu A, Chambrier P, Paindavoine S, Scutt CP (2010). Parallel structural evolution of auxin response factors in the angiosperms. Plant Journal.

[ref-12] Goetz M, Hooper LC, Johnson SD, Rodrigues J, Vivian-Smith A, Koltunow AM (2007). Expression of aberrant forms of AUXIN RESPONSE FACTOR8 stimulates parthenocarpy in *Arabidopsis* and tomato. Plant Physiology.

[ref-13] Guilfoyle TJ, Hagen G (2007). Auxin response factors. Current Opinion in Plant Biology.

[ref-14] Hagen G, Guilfoyle T (2002). Auxin-responsive gene expression: genes, promoters and regulatory factors. Plant Molecular Biology.

[ref-15] Jain M, Khurana JP (2009). Transcript profiling reveals diverse roles of auxin-responsive genes during reproductive development and abiotic stress in rice. FEBS Journal.

[ref-16] Kalluri UC, DiFazio SP, Brunner AM, Tuskan GA (2007). Genome-wide analysis of Aux/IAA and ARF gene families in Populus trichocarpa. BMC Plant Biology.

[ref-17] Krzywinski M, Schein J, Birol I, Connors J, Gascoyne R, Horsman D, Jones SJ, Marra MA (2009). Circos: an information aesthetic for comparative genomics. Genome Research.

[ref-18] Kumar R, Tyagi AK, Sharma AK (2011). Genome-wide analysis of auxin response factor (ARF) gene family from tomato and analysis of their role in flower and fruit development. Molecular Genetics and Genomics.

[ref-19] Li HF, Ran K, Sun QR (2016). Genome-wide identification and expression analysis of peach auxin response factor gene families. Journal of Plant Biochemistry and Biotechnology.

[ref-20] Li M, Wen J, Guo P, Ke Y, Liu M, Li P, Wu Y, Ran F, Wang M, Li J, Du H (2019). The auxin response factor gene family in allopolyploid Brassica napus. PLOS ONE.

[ref-21] Liu N, Wu S, Van Houten J, Wang Y, Ding B, Fei Z, Clarke TH, Reed JW, van der Knaap E (2014). Down-regulation of AUXIN RESPONSE FACTORS 6 and 8 by microRNA 167 leads to floral development defects and female sterility in tomato. Journal of Experimental Botany.

[ref-22] Livak KJ, Schmittgen T (2001). Analysis of relative gene expression data using real-time quantitative PCR and the 2-ΔΔCT method. Methods.

[ref-23] Lu Q, Li H, Hong Y, Zhang G, Wen S, Li X, Zhou G, Li S, Liu H, Liu H (2018). Genome sequencing and analysis of the peanut B-Genome progenitor (*Arachis ipaensis*). Frontiers in Plant Science.

[ref-24] Maas S, Zouine M, Fu Y, Chateigner-Boutin A-L, Mila I, Frasse P, Wang H, Audran C, Roustan J-P, Bouzayen M (2014). Characterization of the tomato ARF gene family uncovers a multi-levels post-transcriptional regulation including alternative splicing. PLOS ONE.

[ref-25] Mao Z, He S, Xu F, Wei X, Jiang L, Liu Y, Wang W, Li T, Xu P, Du S, Li L, Lian H, Guo T, Yang H-Q (2020). Photoexcited *CRY1* and *phyB* interact directly with *ARF6* and *ARF8* to regulate their DNA-binding activity and auxin-induced hypocotyl elongation in *Arabidopsis*. New Phytologist.

[ref-26] Nagpal P, Ellis CM, Weber H, Ploense SE, Barkawi LS, Guilfoyle TJ, Hagen G, Alonso JM, Cohen JD, Farmer EE, Ecker JR, Reed JW (2005). Auxin response factors ARF6 and ARF8 promote jasmonic acid production and flower maturation. Development.

[ref-27] O’Neill DP, Davidson SE, Clarke VC, Yamauchi Y, Yamaguchi S, Kamiya Y, Reid JB, Ross JJ (2010). Regulation of the gibberellin pathway by auxin and DELLA proteins. Planta.

[ref-28] Okushima Y, Overvoorde PJ, Arima K, Alonso JM, Chan A, Chang C, Ecker JR, Hughes B, Lui A, Nguyen D, Onodera C, Quach H, Smith A, Yu GX, Theologis A (2005). Functional genomic analysis of the AUXIN RESPONSE FACTOR gene family members in *Arabidopsis thaliana*: unique and overlapping functions of ARF7 and ARF19. Plant Cell.

[ref-29] Perrot-Rechenmann C (2010). Cellular responses to auxin: division versus expansion. Cold Spring Harbor Perspectives in Biology.

[ref-30] Reddy DS, Bhatnagar-Mathur P, Cindhuri KS, Sharma KK (2013). Evaluation and validation of reference genes for normalization of quantitative real-time pcr based gene expression studies in peanut. PLOS ONE.

[ref-31] Reeves PH, Ellis CM, Ploense SE, Wu MF, Yadav V, Tholl D, Chetelat A, Haupt I, Kennerley BJ, Hodgens C, Farmer EE, Nagpal P, Reed JW (2012). A regulatory network for coordinated flower maturation. PLOS Genetics.

[ref-32] Sparkes IA, Runions J, Kearns A, Hawes C (2006). Rapid, transient expression of fluorescent fusion proteins in tobacco plants and generation of stably transformed plants. Nature Protocols.

[ref-33] Tabata R, Ikezaki M, Fujibe T, Aida M, Tian C, Ueno Y, Yamamoto KT, Machida Y, Nakamura K, Ishiguro S (2010). Arabidopsis AUXIN RESPONSE FACTOR 6 and 8 regulate jasmonic acid biosynthesis and floral organ development via repression of class 1 *KNOX* genes. Plant and Cell Physiology.

[ref-34] Tang G, Peng Z, Xu P, Li P, Zhu J (2020). Genome-wide identification and expression analysis of auxin response factor gene family in Arachis hypogaea L. Chinese Journal of Oil Crop Sciences.

[ref-35] Tiwari SB, Hagen G, Guilfoyle T (2003). The roles of auxin response factor domains in auxin-responsive transcription. Plant Cell.

[ref-36] Truskina J, Han J, Chrysanthou E, Galvan-Ampudia CS, Lainé S, Brunoud G, Macé J, Bellows S, Legrand J, Bgman AM (2021). A network of transcriptional repressors modulates auxin responses. Nature.

[ref-37] Ulmasov T, Hagen G, Guilfoyle TJ (1999). Dimerization and DNA binding of auxin response factors. The Plant Journal.

[ref-38] Ulmasov T, Murfett J, Hagen G, Guilfoyle TJ (1997). Aux/IAA proteins repress expression of reporter genes containing natural and highly active synthetic auxin response elements. The Plant Cell.

[ref-39] Vert G, Walcher CL, Chory J, Nemhauser JL (2008). Integration of auxin and brassinosteroid pathways by Auxin response factor 2. Proceedings of the National Academy of Sciences of the United States of America.

[ref-40] Wang DK, Pei KM, Fu YP, Sun ZX, Li SJ, Liu HQ, Tang K, Han B, Tao YZ (2007). Genome-wide analysis of the auxin response factors (ARF) gene family in rice (*Oryza sativa*). Gene.

[ref-41] Wang S-X, Shi F-Y, Dong X-X, Li Y-X, Zhang Z-H, Li H (2019). Genome-wide identification and expression analysis of auxin response factor (ARF) gene family in strawberry (*Fragaria vesca*). Journal of Integrative Agriculture.

[ref-42] Wen J, Guo PC, Ke YZ, Liu MM, Li PF, Wu YW, Ran F, Wang MM, Li JN, Du H (2019). The auxin response factor gene family in allopolyploid Brassica napus. PLOS ONE.

[ref-43] Weston DE, Reid JB, Ross JJ (2009). Auxin regulation of gibberellin biosynthesis in the roots of pea (*Pisum sativum*). Functional Plant Biology.

[ref-44] Woodward AW, Bartel B (2005). Auxin: regulation, action, and interaction. Annals of Botany.

[ref-45] Wu MF, Tian Q, Reed JW (2006). Arabidopsis microRNA167 controls patterns of *ARF6* and *ARF8* expression, and regulates both female and male reproduction. Development.

[ref-46] Wu J, Wang FY, Cheng L, Kong FL, Peng Z, Liu SY, Yu XL, Lu G (2011). Identification, isolation and expression analysis of auxin response factor (ARF) genes in *Solanum lycopersicum*. Plant Cell Reports.

[ref-47] Wu B, Wang L, Pan G, Li T, Hao J (2020). Genome-wide characterization and expression analysis of the auxin response factor (ARF) gene family during melon (Cucumis melo L.) fruit development. Protoplasma.

[ref-48] Xing HY, Pudake RN, Guo GG, Xing GF, Hu ZR, Zhang YR, Sun QX, Ni ZF (2011). Genome-wide identification and expression profiling of auxin response factor (ARF) gene family in maize. BMC Genomics.

[ref-49] Yang J, Tian L, Sun MX, Huang XY, Zhu J, Guan YF, Yang JZN (2013). AUXIN RESPONSE FACTOR17 is essential for pollen wall pattern formation in *Arabidopsis*. Plant Physiology.

[ref-50] Yin DM, Ji CM, Ma XL, Li H, Zhang WK, Li S, Liu FY, Zhao KK, Li FP, Li K, Ning LL, He JL, Wang YJ, Zhao F, Xie YL, Zheng HK, Zhang XG, Zhang YJ, Zhang JS (2018). Genome of an allotetraploid wild peanut Arachis monticola: a de novo assembly. Gigascience.

[ref-51] Yin DM, Ji CM, Song QX, Zhang WK, Zhang XG, Zhao KK, Chen CY, Wang CT, He GH, Liang Z, Ma XL, Li ZF, Tang YY, Wang YJ, Li K, Ning LL, Zhang H, Zhao K, Li XM, Yu HY, Lei Y, Wang MC, Ma LM, Zheng HK, Zhang YJ, Zhang JS, Hu W, Chen ZJ (2020). Comparison of arachis monticola with diploid and cultivated tetraploid genomes reveals asymmetric subgenome evolution and improvement of peanut. Advanced Science.

[ref-52] Zhang W, Wang SY, Yu FW, Tang J, Shan X, Bao K, Yu L, Wang H, Fei ZJ, Li JB (2019b). Genome-wide characterization and expression profiling of *SWEET* genes in cabbage (*Brassica oleracea* var. capitata L.) reveal their roles in chilling and clubroot disease responses. BMC Genomics.

[ref-53] Zhang YQ, Zeng ZH, Chen CJ, Li CQ, Xia R, Li JG (2019a). Genome-wide characterization of the auxin response factor (ARF) gene family of litchi (*Litchi chinensis* Sonn.): phylogenetic analysis, miRNA regulation and expression changes during fruit abscission. Peerj.

[ref-54] Zhao YD (2010). Auxin biosynthesis and its role in plant development. Annual Review of Plant Biology.

[ref-55] Zhao Y, Wong Q, Ma H, Song J, Yuan J (2016). Genome-wide identification and bioinformatics analysis of *ARF* gene family in *Setaria Italica*. Journal of Plant Genetic Resources.

[ref-56] Zhuang WJ, Chen H, Yang M, Wang JP, Pandey MK, Zhang C, Chang WC, Zhang LS, Zhang XT, Tang RH, Garg V, Wang XJ, Tang HB, Chow CN, Wang JP, Deng Y, Wang DP, Khan AW, Yang Q, Cai TC, Bajaj P, Wu KC, Guo BZ, Zhang XY, Li JJ, Liang F, Hu J, Liao BS, Liu SY, Chitikineni A, Yan HS, Zheng YX, Shan SH, Liu QZ, Xie DY, Wang ZY, Khan SA, Ali N, Zhao CZ, Li XG, Luo ZL, Zhang SB, Zhuang RR, Peng Z, Wang SY, Mamadou G, Zhuang YH, Zhao ZF, Yu WC, Xiong FQ, Quan WP, Yuan M, Li Y, Zou HS, Xia H, Zha L, Fan JP, Yu JG, Xie WP, Yuan JQ, Chen K, Zhao SS, Chu WT, Chen YT, Sun PC, Meng FB, Zhuo T, Zhao YH, Li CJ, He GH, Zhao YL, Wang CC, Kavikishor PB, Pan RL, Paterson AH, Wang XY, Ming R, Varshney RK (2019). The genome of cultivated peanut provides insight into legume karyotypes, polyploid evolution and crop domestication. Nature Genetics.

