# Supplementary figures and images for: Genome-wide identification and expression analysis of auxin response factors in peanut (Arachis hypogaea L.)
Source: PeerJ. 2021 Oct 21;9:e12319. doi: 10.7717/peerj.12319 (PMC8542371; doi:10.7717/peerj.12319)

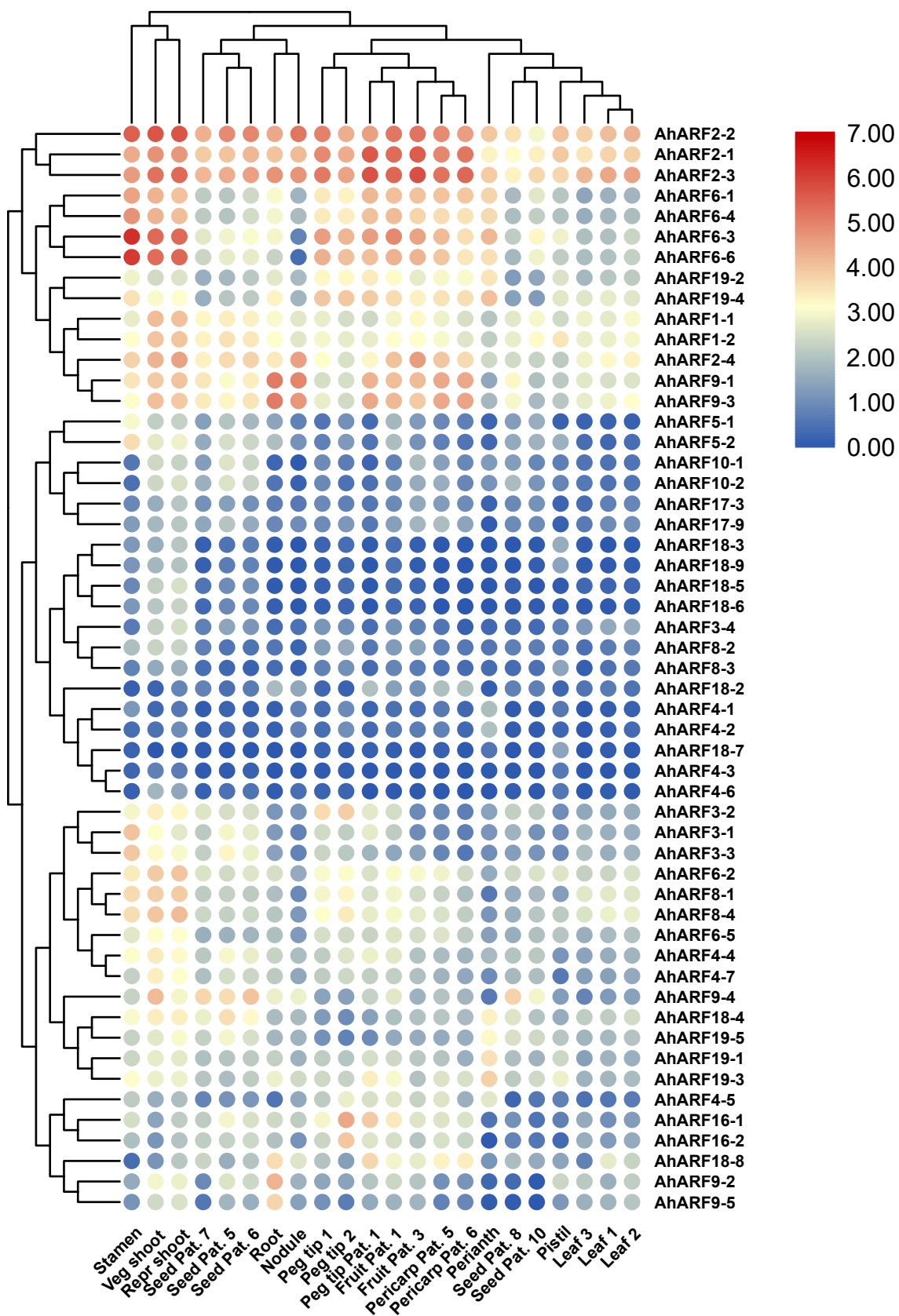

Supplement: Supplemental Information 1 — The dates are from an expression atlas (Clevenger et al., 2016), Bar showed log2 (FPKM) colored blue to red. Information on 22 tissues has been provided in Additional file 2: Table S2. Fragments per Kilobase per Million mapped reads (FPKM) values of the AhARF genes are listed in Additional file 2: Table S6. [file peerj-09-12319-s001.pdf]

**AhARF 6-1**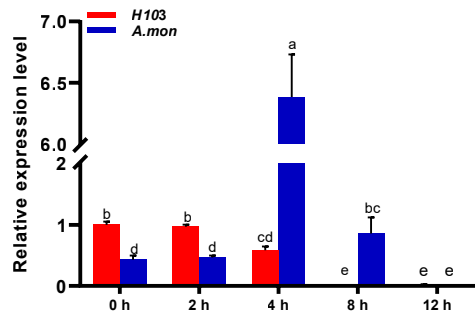**AhARF 6-2**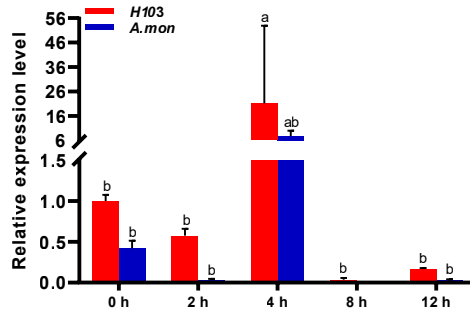**AhARF 6-3**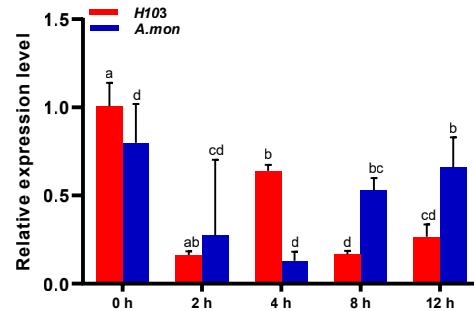**AhARF 6-4**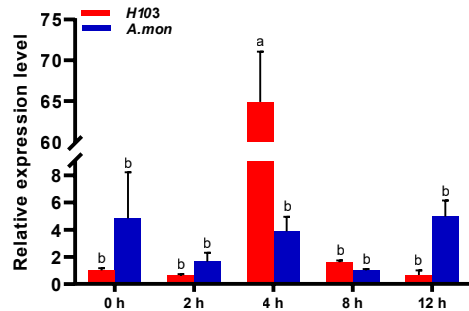**AhARF 6-5**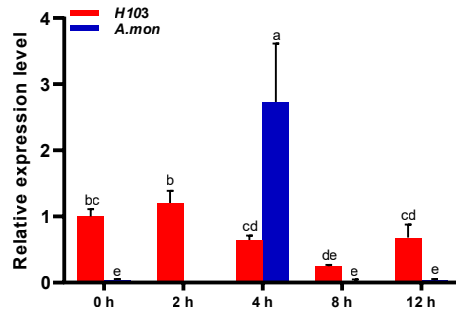**AhARF 6-6**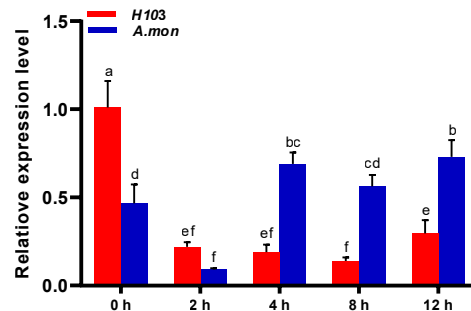

Supplement: Supplemental Information 2 — qRT-PCR analysis of AhARF6 transcript levels was performed at the fifth leaf stage of plants treated with NAA for 0, 2, 4, 8, and 12 h. Values are means ± SD (n = 3). Different lowercase letters denote significant differences between the any two stages (P < 0.05, one-way ANOVA and Tukey’s test for multiple comparisons). [file peerj-09-12319-s002.pdf]

**AhARF 6-1**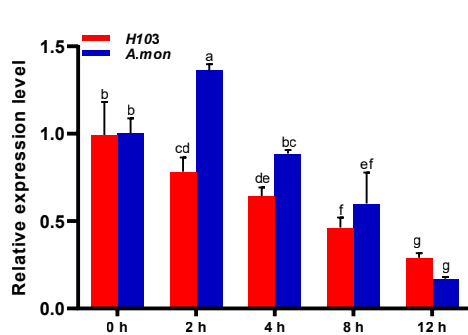**AhARF 6-2**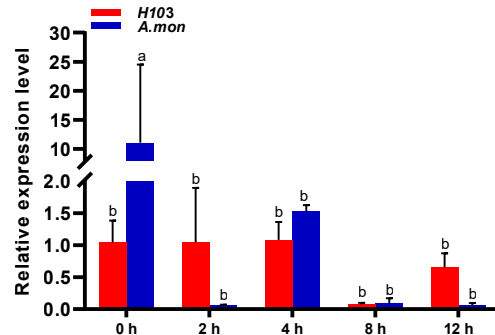**AhARF 6-3**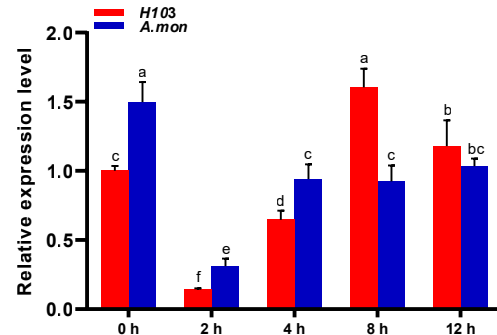**AhARF 6-4**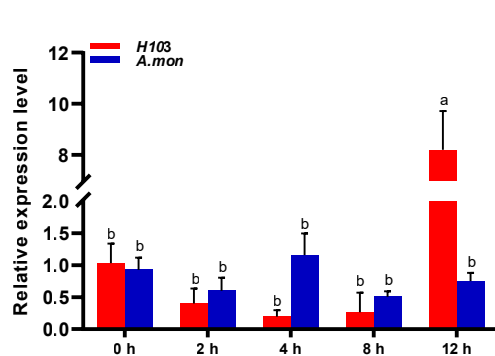**AhARF 6-5**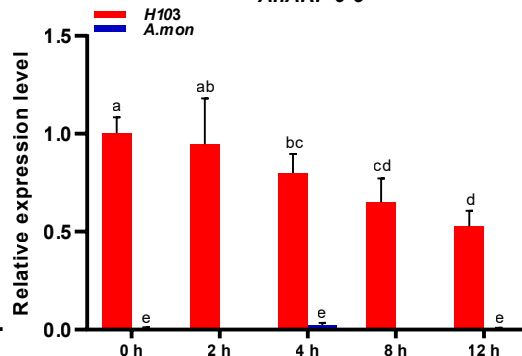**AhARF 6-6**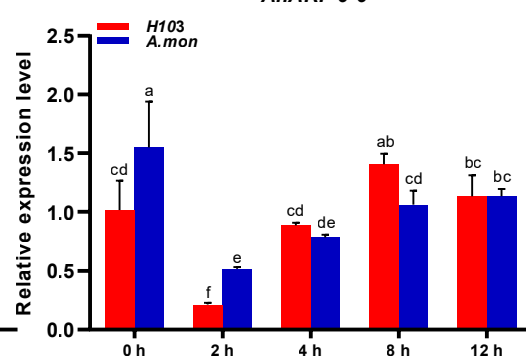

Supplement: Supplemental Information 3 — qRT-PCR analysis of AhARF6 transcript levels was performed at the fifth leaf stage of plants treated with NAA for 0, 2, 4, 8, and 12 h. Values are means ± SD (n = 3). Different lowercase letters denote significant differences between the any two stages (P < 0.05, one-way ANOVA and Tukey’s test for multiple comparisons). [file peerj-09-12319-s003.pdf]
